# Transition From Microscopic to Endoscopic Transsphenoidal Surgery: Challenges, Advantages, and Early Surgical Outcomes

**DOI:** 10.7759/cureus.105997

**Published:** 2026-03-27

**Authors:** Recai Engin, Fatih Tomakin, Cem Demirel, Vaner Köksal, Esra Yılmaz, Hande Arslan, Doğukan Özdemir

**Affiliations:** 1 Department of Neurosurgery, Samsun University Faculty of Medicine, Samsun, TUR; 2 Department of Otolaryngology-Head and Neck Surgery, Samsun University Faculty of Medicine, Samsun, TUR; 3 Department of Otolaryngology-Head and Neck Surgery, Samsun Education and Research Hospital, Samsun, TUR

**Keywords:** endoscopic endonasal surgery, learning curve, pitnet, pituitary tumor, transsphenoidal approach

## Abstract

Objective: The aim of this study was to evaluate the early clinical and surgical outcomes during the transition from microscopic transsphenoidal surgery to the endoscopic endonasal transsphenoidal approach.

Materials and methods: This retrospective study included the first 10 consecutive patients with pituitary tumors who were operated on using the endoscopic endonasal transsphenoidal approach by a surgical team experienced in microscopic transsphenoidal surgery. All procedures were performed by the same multidisciplinary team using a four-hand technique in collaboration with an otorhinolaryngologist. Patients were evaluated in terms of demographic characteristics, tumor type and size, cavernous sinus invasion, extent of resection, complications, and early clinical outcomes.

Results: Seven patients were male, and three were female, with a mean age of 52.5±13.1 years. Histopathological examination revealed non-functioning pituitary neuroendocrine tumor (pitNET) in three patients, gonadotroph pitNET in four patients, corticotroph pitNET in two patients, and somatotroph pitNET in one patient. Cavernous sinus invasion was observed in four patients. Early postoperative imaging demonstrated residual tumor in two patients, whereas gross total resection was achieved in eight patients. Intraoperative cerebrospinal fluid leakage occurred in three patients; one patient required additional surgical intervention for a postoperative cerebrospinal fluid fistula. No major vascular complications were observed. Permanent diabetes insipidus developed in one patient. The mean operative time was 4.5±0.6 hours, and the mean intraoperative blood loss was 178±15.5 mL.

Conclusion: During the early phase of the transition from microscopic to endoscopic endonasal transsphenoidal surgery, acceptable surgical outcomes can be achieved with appropriate patient selection and a multidisciplinary team approach. These findings suggest that the endoscopic technique can be safely implemented even in the early stages of the learning curve.

## Introduction

The primary goal of pituitary surgery is to achieve maximal safe resection of sellar pathologies. This approach enables the effective control of hormone hypersecretion, alleviation of symptoms related to mass effect, and particularly decompression of the optic pathways while also contributing to a reduced risk of tumor recurrence during long-term follow-up [1]. The selection of an appropriate surgical approach depends on multiple variables. Sellar anatomy, the degree of sphenoid sinus pneumatization, the anatomical course of the carotid arteries, and the extent and growth pattern of the tumor are among the key factors influencing surgical planning. Careful, patient-specific evaluation of these parameters is essential to achieve optimal surgical outcomes [2]. However, despite these well-established considerations, there is limited data in the literature regarding the early clinical experience and outcomes during the transition from microscopic to endoscopic transsphenoidal surgery, particularly in centers with an established microscopic surgical background.

Endoscopic endonasal transsphenoidal surgery has become the gold standard approach for pituitary tumor resection by offering a minimally invasive corridor with low morbidity [3-5]. The traditional microscopic transsphenoidal approach remains a well-established technique for pituitary tumor removal and provides excellent depth perception, offering the neurosurgeon a sense of security during surgery. However, this technique has inherent limitations in visualizing the surgical field and may fail to provide adequate panoramic visualization, particularly in the suprasellar and lateral extensions of the tumor [2]. In contrast, the endoscope allows for a wider surgical field of view and has been associated with improved exposure and fewer intraoperative complications [1].

When operating under an endoscope, the neurosurgeon has to adapt not only to the new tool but also to a new workspace: the endonasal approach without a speculum and without stereoscopic vision [6]. Proponents of the traditional microscopic technique argue that it offers a three-dimensional visualization of the tumor with the binocular operating microscope and, thus, the ability to operate in a three-dimensional space, something that conventional two-dimensional endoscopic systems do not offer due to their lack of depth perception, although recently developed three-dimensional endoscopic systems may partially overcome this limitation [7].

In this study, we aimed to share our early surgical experience during the transition from microscopic transsphenoidal surgery to the endoscopic endonasal transsphenoidal technique in pituitary surgery. The technical adaptation process encountered during the integration of the endoscopic approach into routine surgical practice, the challenges faced by the surgeon, and the advantages that became evident over time are discussed. Additionally, by evaluating early outcomes obtained from a limited number of cases, this study seeks to provide practical insights into the safe applicability of the endoscopic approach during the transition period.

## Materials and methods

This study is a descriptive retrospective clinical case series evaluating patients who underwent endoscopic endonasal transsphenoidal pituitary surgery at the Department of Neurosurgery of Samsun University City Hospital in Samsun, Turkey, between July 1, 2025, and January 1, 2026. The study includes patients operated on during the transition period from the microscopic transsphenoidal approach to the endoscopic endonasal technique. The study protocol was approved by the Samsun University Non-interventional Clinical Research Ethics Committee, and all diagnostic and therapeutic procedures were conducted in accordance with the principles of the Declaration of Helsinki. The ethics committee number is GOKAEK 2026/1/7.

The first 10 consecutive patients with pituitary tumors who were operated on using the endoscopic endonasal transsphenoidal approach were included in the study. Due to the descriptive nature of the study and the small sample size (n=10), no inferential statistical analyses were performed, and the data were presented using descriptive statistics only. All surgical procedures were performed by the same surgical team. Patients with a history of prior pituitary surgery, those who underwent revision surgery, or patients who had received preoperative radiotherapy were excluded from the study. These patients had been operated on using the microscopic transseptal transsphenoidal technique. This exclusion was intended to allow a more homogeneous evaluation of outcomes related to the early learning phase of endoscopic surgery.

All patients underwent comprehensive preoperative neurological, endocrinological, and ophthalmological evaluations. In the preoperative period, paranasal sinus computed tomography (CT) was performed in all cases, with particular attention to nasal cavity and sphenoid sinus anatomy for surgical planning. Magnetic resonance imaging (MRI) was used to assess tumor size, sellar localization, relationship with the sphenoid sinus, and suprasellar extension. The extent of resection and the presence of residual tumor were analyzed based on early postoperative MRI findings.

In all patients, routine serum and urine electrolyte levels as well as pituitary hormone profiles were evaluated preoperatively by the endocrinology department. Ophthalmological assessment, including Humphrey visual field testing, was performed by ophthalmologists. Rhinological examinations were conducted endoscopically by rhinologists, and paranasal sinus CT scans were reviewed in all patients. Radiological evaluation was based on MRI findings, and tumors were analyzed according to the Hardy and Knosp classification systems.

All surgical procedures were performed using an endoscopic endonasal transsphenoidal approach. Operations were conducted using a four-hand technique in collaboration with an otorhinolaryngology specialist. After exposure of the sphenoid sinus, access to the sellar floor was achieved, and the localization of the carotid arteries was confirmed using intraoperative Doppler ultrasonography prior to dural opening. Tumor excision was performed under neuronavigation guidance, with particular attention to maintaining anatomical orientation and ensuring the safety of critical neurovascular structures. Different endoscopic viewing angles were utilized when necessary, depending on the extent and growth pattern of the tumor.

In cases where intraoperative cerebrospinal fluid (CSF) leakage occurred, reconstruction was performed according to the grade of leakage and the size of the defect. A vascularized nasoseptal flap (Hadad flap) was used in patients with high-flow CSF leakage, whereas simpler reconstruction techniques were preferred in cases with low-risk leakage or no observed CSF leak. Patients were closely monitored postoperatively for the development of CSF fistula and other surgical complications.

Demographic characteristics, operative times, extent of resection, intraoperative and postoperative CSF leakage, need for Hadad flap reconstruction, early postoperative complications, and length of hospital stay were obtained from patient medical records. Due to the descriptive nature of the study and the limited number of patients, no advanced comparative statistical analyses were performed. Data were presented using descriptive statistics, with continuous variables expressed as mean and standard deviation and categorical variables as numbers and percentages.

## Results

Of the 10 patients included in the study, seven were male, and three were female. Patient ages ranged from 26 to 70 years, with a mean age of 52.5±13.1 years (Table 1). Histopathological examination revealed three non-functioning pituitary neuroendocrine tumors (pitNETs), four gonadotroph pitNETs, two corticotroph pitNETs, and one somatotroph pitNET. Tumor sizes ranged from 11 mm to 43 mm (11, 12, 16, 19, 22, 23, 26, 33, 36, and 43 mm, respectively). Cavernous sinus invasion was identified in four patients.

**Table 1:** Baseline characteristics of patients and pituitary tumors Data are presented as number (n) and percentage (%) for categorical variables and mean±standard deviation (SD) for continuous variables. No inferential statistical tests were performed due to the descriptive nature of the study and the small sample size. pitNET: pituitary neuroendocrine tumor

Variable	Category	n (%) or mean±SD
Number of patients	-	10
Age (years)	-	52.5±13.1
	Range	26-70
Sex	Male	7 (70%)
	Female	3 (30%)
Preoperative Humphrey visual field	Normal	3 (30%)
	Visual field defect	7 (70%)
Postoperative Humphrey visual field	Normal	3 (30%)
	Improved	4 (40%)
	Unchanged	3 (30%)
Histopathological diagnosis	Non-functioning pitNET	3 (30%)
	Gonadotroph pitNET	4 (40%)
	Corticotroph pitNET	2 (20%)
	Somatotroph pitNET	1 (10%)
Tumor size	10-20 mm	4 (40%)
	20-30 mm	3 (30%)
	30-40 mm	2 (20%)
	>40 mm	1 (10%)

Early postoperative MRI evaluation demonstrated residual tumor in two patients (Table 2). One of these cases involved a 33-mm gonadotroph pitNET with a residual tumor measuring 18 mm. The other patient had a 22-mm non-functioning pitNET with a residual tumor measuring 9 mm. In both cases, resection was intentionally limited due to tumor adherence to surrounding critical structures, with surgical safety prioritized. Radiological total resection was achieved in the remaining eight patients.

**Table 2:** Surgical outcomes and perioperative complications following endoscopic endonasal transsphenoidal surgery Data are presented as number (n) and percentage (%) for categorical variables and mean±standard deviation (SD) for continuous variables. No inferential statistical tests were performed due to the descriptive nature of the study and the small sample size. CSF: cerebrospinal fluid; MRI: magnetic resonance imaging

Variable	Category	n (%) or mean±SD
Operative time (hours)	-	4.5±0.6
Intraoperative blood loss (mL)	-	178±15.5
Major bleeding/vascular injury	Yes	0 (0%)
	No	10 (100%)
Intraoperative CSF leak	Yes	3 (30%)
	No	7 (70%)
Reconstruction technique	Fat graft + fibrin glue + cartilage + vascularized nasoseptal flap	3 (30%)
	Fat graft + fibrin glue + cartilage	7 (70%)
Early postoperative MRI	Residual tumor present	2 (20%)
	No residual tumor	8 (80%)
Postoperative complications	Transient polyuria	4 (40%)
	Diabetes insipidus	1 (10%)
	Postoperative rhinorrhea	1 (10%)
	Lumbar drainage requirement	1 (10%)
	Pneumocephalus	1 (10%)
	Endoscopic CSF leak repair during follow-up	1 (10%)
	Epistaxis	1 (10%)
	None	4 (40%)
Hospital stay (days)	-	6.1±3.6
Follow-up (months)	-	4.0±1.2

Preoperatively, three patients had no visual field deficits. With the exception of the patient with an 18-mm residual tumor, all patients reported subjective improvement in visual function postoperatively. Objective evaluation using Humphrey visual field testing showed improvement in four patients compared with preoperative findings, while no significant change was observed in three patients. The patient with a residual tumor and no visual field improvement was scheduled for reoperation.

Intraoperative CSF leakage occurred in three patients. In these cases, reconstruction was performed using a combination of fat graft, fibrin sealant, cartilage, and a vascularized nasoseptal flap (Hadad flap). In patients without CSF leakage, closure was achieved using fat graft, fibrin sealant, and cartilage. One patient diagnosed with a 36-mm corticotroph pitNET developed rhinorrhea during the first postoperative week and underwent lumbar drainage. Subsequently, pneumocephalus developed, and the patient required endoscopic CSF fistula repair.

The mean operative time was 4.5±0.6 hours, and the mean intraoperative blood loss was 178±15.5 mL. All patients were monitored in the surgical intensive care unit for one day postoperatively, during which biochemical parameters and hourly urine output were closely observed. All patients were evaluated postoperatively by an endocrinologist. Transient polyuria was defined as increased urine output without associated electrolyte imbalance or need for treatment, resolving spontaneously within 48 hours. Diabetes insipidus was diagnosed in cases with persistent polyuria requiring antidiuretic hormone analogue therapy. One patient with a corticotroph pitNET developed postoperative diabetes insipidus and was treated with an antidiuretic hormone analogue. Although polyuria was observed in four patients during the first two postoperative days, no biochemical abnormalities were detected, and no treatment was required; this condition resolved spontaneously after the second day.

No major intraoperative bleeding or vascular injury occurred in any patient. One patient developed epistaxis during the first postoperative week, which was successfully managed with nasal packing. Except for the patient who required reoperation for CSF fistula repair, the remaining nine patients were discharged on postoperative day 5. The length of hospital stay for the patient who underwent CSF fistula repair was 16 days. The mean follow-up period was 4±1.2 months.

## Discussion

In this study, the early clinical and surgical outcomes of the transition from microscopic transsphenoidal surgery to the endoscopic endonasal transsphenoidal approach were evaluated. The literature has consistently reported that the endoscopic approach provides a wider surgical field of view compared with the microscopic technique, offers advantages in the assessment of suprasellar and parasellar extensions, and achieves similar or superior resection rates with an acceptable complication profile [1,2].

In our series, the wide range of tumor sizes and the presence of cavernous sinus invasion in a subset of patients indicate that the surgical experience during the transition period encompassed lesions with varying degrees of complexity. The detection of residual tumor in two patients during early postoperative evaluation may be interpreted as a finding potentially associated with the early phase of the endoscopic learning curve; however, given the limited sample size, this observation should be interpreted with caution. It has previously been reported that total resection may not always be achievable, particularly in cases with cavernous sinus invasion [1,7].

Among the major limitations of endoscopic surgery are the lack of stereoscopic vision, the risk of instrument collision within the surgical field, frequent blurring of the visual field due to contact of the endoscope lens with blood or tissue, and the requirement for close coordination between two surgeons. These factors may contribute to a steeper learning curve, especially during the transition period [3,6]. However, it has also been reported that with increasing experience, these disadvantages can be largely overcome and the panoramic view provided by the endoscopic approach enhances surgical control [1,6].

While the operation times reported in early endoscopic surgery series in the literature were less than three hours, they were slightly longer in our series [8]. Previous meta-analyses have demonstrated that operative time tends to be relatively prolonged during the early phases of the learning curve but decreases significantly as surgical experience increases [4]. The absence of major vascular injury in our series may suggest a potential contribution of intraoperative Doppler ultrasonography and neuronavigation to surgical safety, although definitive conclusions cannot be drawn from this limited series. In line with the literature, intraoperative blood loss was low [9], which may be attributed to the utilization of anatomical corridors inherent to endoscopic pituitary surgery.

Shorter hospital stays following endoscopic pituitary surgery have been reported in the literature, with an average length of stay of 2-3 days [10]. In our study, the mean length of hospitalization was slightly longer compared with these reports. This difference may be related to closer postoperative monitoring during the transition phase, although institutional practice patterns and patient selection may also have contributed during the transition phase to endoscopic surgery. Nevertheless, studies reporting average hospital stays exceeding five days are also available in the literature [1].

The incidence of permanent diabetes insipidus after endoscopic pituitary surgery has been reported to range between 4% and 11% [2,8,11,12]. In our series, one patient (10%) developed permanent diabetes insipidus, which was managed medically by endocrinologists.

While intraoperative CSF leakage was observed in three cases, postoperative rhinorrhea occurred in only one patient (10%). The literature reports CSF fistula rates below 10% in endoscopic surgery [8,13]. However, during the early stages of endonasal experience, CSF leakage rates ranging from 10% to 40% have been described [14,15]. Qureshi et al. reported a CSF fistula rate of 33% in the first nine patients (early group), compared with 7.4% in the subsequent 68 patients (late group) [11]. The gradual reduction in CSF fistula rates has been attributed to increasing surgeon experience and improvements in reconstruction techniques. In our study, closure in patients with intraoperative CSF leakage was performed using fat grafts, fibrin sealant, cartilage, and a vascularized nasoseptal flap. This reconstruction strategy is widely reported in the literature as an effective method for preventing postoperative CSF leakage [16]. Despite this, one patient required secondary surgical repair for CSF fistula, which may be considered within the range reported in early endoscopic experience, although the small sample size limits definitive interpretation.

The most important parameters influencing total resection of pituitary tumors include tumor consistency, invasion of the internal carotid artery and cavernous sinus, and the presence of suprasellar extension [17-19]. In our study, evaluation of the two patients with residual tumor suggests that incomplete resection was primarily related to tumor consistency and cavernous sinus invasion. Conversely, a 43-mm tumor with suprasellar extension was totally resected due to its soft consistency. Krishna et al. reported a total resection rate of 81.39% in endoscopic pituitary surgery [2].

Although our findings provide preliminary insight into the early phase of transition to endoscopic transsphenoidal surgery, several limitations must be acknowledged. The small sample size and descriptive design preclude definitive conclusions regarding the learning curve or comparative safety of the technique. In addition, the absence of a microscopic control group and the lack of a structured comparison between early and later cases limit the ability to draw causal inferences. Furthermore, the study cohort may reflect a degree of case selection during the transition period; therefore, the observed outcomes may not be generalizable to more complex sellar pathologies. Finally, endocrine outcomes were not analyzed in detail due to the short follow-up period and the primary focus on early surgical results.

Limitations

This study has several limitations. First, the sample size is relatively small, reflecting the early phase of the transition from microscopic to endoscopic transsphenoidal surgery. Second, the retrospective design may introduce inherent biases related to patient selection and data collection. Third, the follow-up duration is relatively short and therefore does not allow for a comprehensive evaluation of long-term surgical outcomes, endocrine remission rates, or tumor recurrence.

Importantly, the study does not include a direct comparison with a microscopic transsphenoidal cohort, which limits the ability to determine whether the transition to the endoscopic technique resulted in improved, equivalent, or inferior outcomes. In addition, the absence of a control group and the single-center nature of the study may further limit the generalizability of the findings. It should also be considered that case selection during the early transition period may have been influenced by a preference for more favorable or less complex lesions, which may introduce selection bias.

Finally, due to the descriptive nature of the study and the limited number of patients, no inferential statistical analyses were performed. Future studies, including larger patient cohorts, comparative designs, and longer follow-up periods, are needed to better assess the long-term effectiveness and safety of the endoscopic endonasal approach during the transition period.

## Conclusions

This study presents early clinical experience during the transition from microscopic transsphenoidal surgery to the endoscopic endonasal transsphenoidal approach. The findings suggest that, in carefully selected patients and with a multidisciplinary surgical team approach, the endoscopic technique is feasible and may yield acceptable short-term outcomes during the initial phase of the learning curve. Safety-oriented surgical planning may contribute to maintaining complication rates within acceptable limits; however, these observations should be interpreted with caution given the limited sample size and short follow-up duration. Larger patient series and long-term follow-up data are required to more clearly define the overall safety, effectiveness, and durability of this technical transition.
